# A Level Set Based Framework for Quantitative Evaluation of Breast Tissue Density from MRI Data

**DOI:** 10.1371/journal.pone.0112709

**Published:** 2014-11-25

**Authors:** Tatyana Ivanovska, René Laqua, Lei Wang, Volkmar Liebscher, Henry Völzke, Katrin Hegenscheid

**Affiliations:** 1 Institute of Community Medicine, University Medicine Greifswald, Greifswald, Germany; 2 Institute of Diagnostic Radiology and Neuroradiology, University Medicine Greifswald, Greifswald, Germany; 3 Fraunhofer Institute for Medical Image Computing MEVIS, Bremen, Germany; 4 Institute of Mathematics and Informatics, Ernst-Moritz-Arndt University, Greifswald, Germany; University of Zurich, Switzerland

## Abstract

Breast density is a risk factor associated with the development of breast cancer. Usually, breast density is assessed on two dimensional (2D) mammograms using the American College of Radiology (ACR) classification. Magnetic resonance imaging (MRI) is a non-radiation based examination method, which offers a three dimensional (3D) alternative to classical 2D mammograms. We propose a new framework for automated breast density calculation on MRI data. Our framework consists of three steps. First, a recently developed method for simultaneous intensity inhomogeneity correction and breast tissue and parenchyma segmentation is applied. Second, the obtained breast component is extracted, and the breast-air and breast-body boundaries are refined. Finally, the fibroglandular/parenchymal tissue volume is extracted from the breast volume. The framework was tested on 37 randomly selected MR mammographies. All images were acquired on a 1.5T MR scanner using an axial, T1-weighted time-resolved angiography with stochastic trajectories sequence. The results were compared to manually obtained groundtruth. Dice's Similarity Coefficient (DSC) as well as Bland-Altman plots were used as the main tools for evaluation of similarity between automatic and manual segmentations. The average Dice's Similarity Coefficient values were 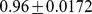 and 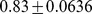 for breast and parenchymal volumes, respectively. Bland-Altman plots showed the mean bias (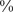) 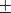 standard deviation equal 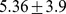 for breast volumes and 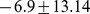 for parenchyma volumes. The automated framework produced sufficient results and has the potential to be applied for the analysis of breast volume and breast density of numerous data in clinical and research settings.

## Introduction

The mammographic breast density is defined as the area of dense tissue on a mammogram divided by the total area of the imaged breast (percent mammographic density). A systematic meta-analysis using data of more than 14 000 women with breast cancer and 226 000 women without breast cancer from 42 studies showed that increased breast density of more than 50% was consistently associated with an increased risk of breast cancer [1]. Further, various case-control studies within large, prospective cohort studies from Europe, the United States and Canada showed a four to five times increase in breast cancer risk in women with dense breasts [2]–[10]. Breast density is usually estimated using the classification system of the Breast Imaging Reporting and Data System (BI-RADS) by the American College of Radiology [11].

Commonly, breast density is evaluated on two dimensional (2D) X-ray mammograms, which introduces substantial measurement errors, since the breast is a three dimensional (3D) structure. Magnetic Resonance Imaging (MRI) mammograms (MRM) have a non-ionizing nature and strong soft tissue contrast between fibroglandular (parenchymal) and fatty tissue. Therefore, MRM provide an alternative to the classical approach especially in research setting, where the application of X-ray is not ethically justified. Moreover, the 3D breast density evaluation should reduce the measurement errors, which appear in 2D case. The quantitative 3D breast density evaluation, executed by the user manually, is a laborious, observer-dependent, and extremely time-consuming process. Therefore, full or partial automation of the 3D analysis of breast is required.

Recently, a few approaches for automated breast density evaluation have been developed [12]–[18]. However, most of these methods consist of numerous processing steps, which may serve as an additional source of errors, or require an extensive user interaction (e.g., the methods of Klifa et al. [12], Nie et al. [14], Lin et al. [13], and Wang et al. [15]). Some methods require training on a significant number of manually segmented datasets (e.g., the atlas-based approaches of Gubern-Merida et al. [16] and Gallego Ortiz and Martel [17]), or have been developed for a specific data sequence (e.g., the approach designed for sagittal breast images by Wu et al. [18]). Moreover, all these methods have been designed for MRI sequences that do not have such strong inhomogeneities as the ones used in our study.

Therefore, the objectives of this study are to develop an automated framework for breast density estimation that a) does not extensively involve the user, b) is suitable for data with strong intensity inhomogeneities, c) does not have numerous processing and correction steps, since each step might introduce additional errors. We propose a method that allows us to segment total breast volume (BV), fibroglandular (parenchymal) tissue volume (PV), and correct bias field in one pass. The main step is the recently proposed level set based method for simultaneous intensity inhomogeneity correction and segmentation [19] followed by a boundary refinement procedure. The approach requires only minimal user interaction, and the methods parameters are pre-selected for different ACR groups.

## Materials and Methods

### Study population

This study was a subproject of the population-based Study of Health in Pomerania (SHIP). SHIP is conducted in the Northeast German federal state of Mecklenburg-Western Pomerania [20]. The general objective of the SHIP is to estimate the prevalence and incidence of common diseases and corresponding risk factors. A whole-body MRI including a contrast-enhanced 3D MRM is part of the examination protocol of the previous examination waves SHIP-2 and SHIP-Trend-0 [21]. One specific aim is to assess breast density on 3D MRM and to associate it with several risk factors, clinical examination results, metabolic and genome wide analysis. Between 2008 and 2012, a total of 3372 participants (mean age 53 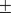 14 (standard deviation) years) underwent a standardized whole-body MRI examination protocol. Of the 1 717 (50.9%) women 1 433 decided to participate in a 3D MRM examination. The SHIP was conducted as approved by the local Institutional Review Board at Greifswald University Hospital. Written informed consent was obtained separately for study inclusion and MR imaging.

### MR Image Acquisition

**Figure 1:**
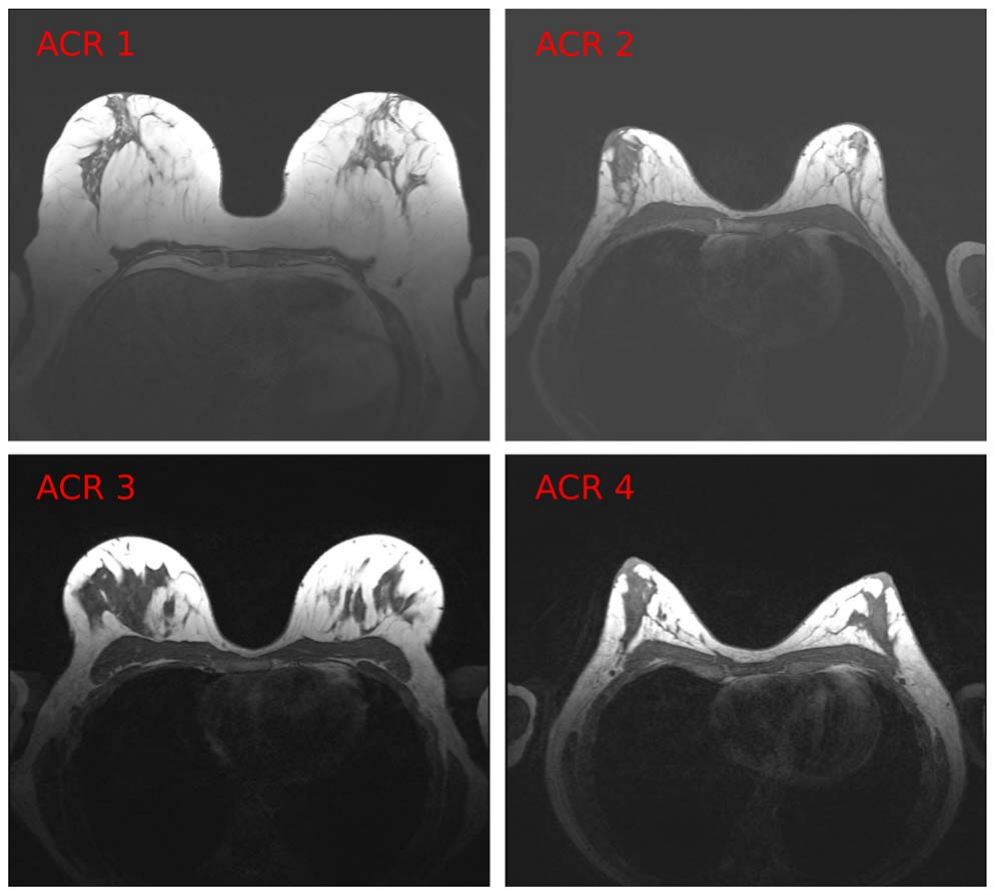
Example slices from 4 different datasets (correspondent to 4 subjects). Each dataset belongs to a different ACR group.

Breast MR imaging was performed at 1.5 Tesla on a whole-body MR imager (Magnetom Avanto; Siemens Medical Solutions, Erlangen, Germany). The woman was placed in prone position with the uncompressed breasts suspended in a circularly polarized bilateral breast phased-array four-channel receiver coil (Siemens Medical Solutions). The following images were acquired after obtaining localizer images: an axial turbo-inversion recovery magnitude sequence (TIRM) (5800/56 [repetition time msec/echo time msec]; 150° flip angle; 340 mm field of view; 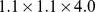 mm voxels), an axial T2-weighted non-fat-suppressed sequence (4660/67 [repetition time msec/echo time msec]; 180° flip angle; 340 mm field of view; 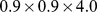 mm voxels), a fat-suppressed, diffusion-weighted echo-planar sequence (7900/91; 340 mm field of view; 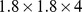 mm voxels; with b values of 50, 200, 500, 800, and 1,000 sec/mm2), and an unenhanced non-fat-suppressed three-dimensional T1-weighted time-resolved sequence with stochastic trajectories (TWIST) (8.86/4.51; 25° flip angle; 340 mm field of view; 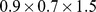 mm voxels) in the axial plane. For dynamic contrast-enhanced MR mammography after acquisition of the first unenhanced TWIST sequence, an intravenous gadobutrol bolus (Gadovist, Bayer Healthcare, Leverkusen, Germany) was administered with a power injector at a dose of 0.1 mmol/kg body weight at a rate of 1.0 mL/s, followed by a saline flush (20 mL) injected at the same rate. The TWIST sequence was repeated five times without time gaps. Each sequence took 58.27 seconds. Image subtraction was performed automatically by the scanner system. An experienced radiologist (KH, with more than eight years experience in MR mammography) read all MRMs for the presence of breast lesions and classified breast density into four groups for all examinations according to the BI-RADS classification system [11]. After exclusion of 125 cases due to breast surgery/implants (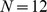) or breast lesions (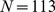) 37 cases were selected randomly from the four breast density groups. Four example 2D slices correspondent to four different subjects are shown in Figure 1.

#### MR Data Artefacts

**Figure 2:**
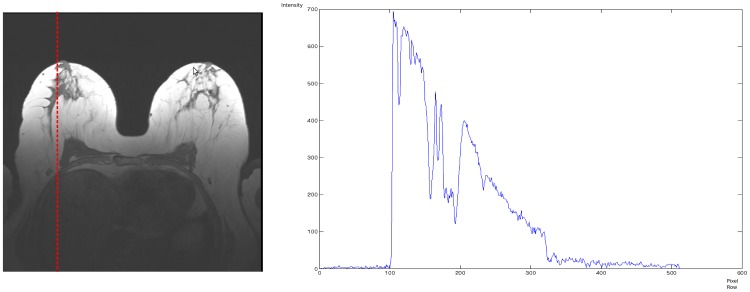
Intensity profile along the line, marked red in the example slice. The intensities of the same tissue (for example, the fatty part) in the upper slice part are about 700, and in the lower breast part they are close to 100.

Our data exhibit quite strong intensity inhomogeneities, which come from different sources. First, there is a clear gradient from the breast nipple in the direction of the heart, which can be explained by increasing offset from the receiving coil, located in the front of the chest. To demonstrate the intensity gradient present in the image, we depict the intensity profile along a line in Figure 2. The image starts from a rather low background values close to zero and, then, a smooth intensity decrease appears. The intensities of the same tissue (for example, the fatty tissue) near the nipple are much higher (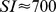) than in the lower part near to chest (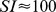).

**Figure 3:**
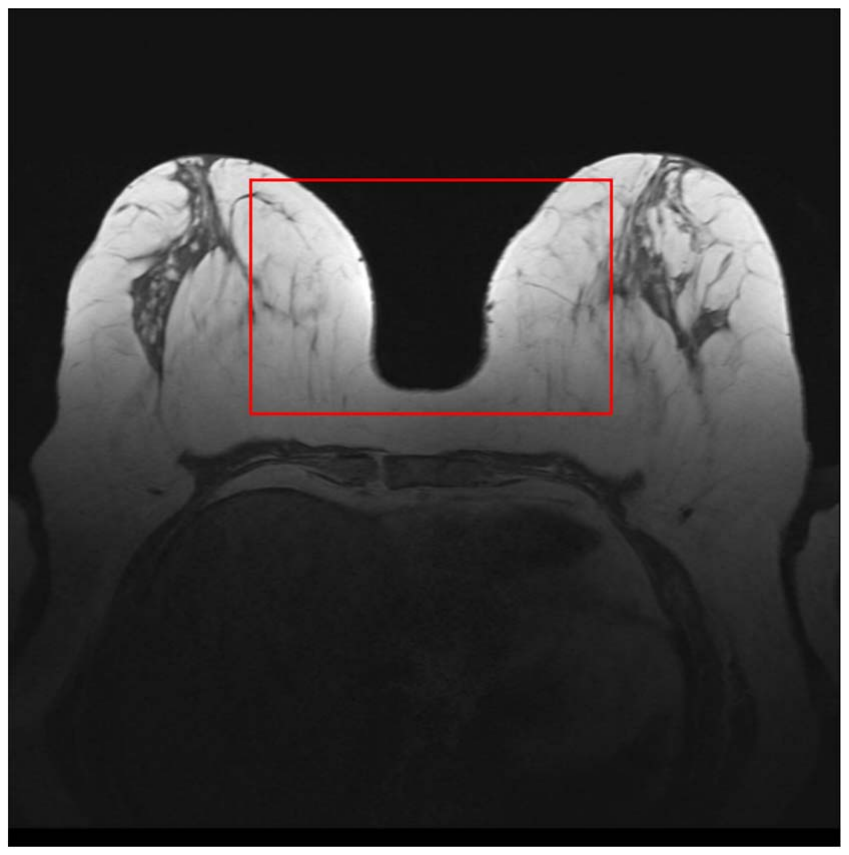
Another intensity inhomogeneity example. The intensities of the breast tissue close to the breast air boundaries (marked by the red rectangle) have higher values than the intensities of the breast tissue located in the middle of the breast.

Second, there is a left-right gradient in breast tissue, which is caused by the radiofrequency (B1) field [22]. In Figure 3, one can observe that the breast tissue close to the breast-air boundary (inside the red rectangle) has slightly higher intensity values than the ones in the middle of the breast. Such artefacts usually represent a significant problem for intensity-based segmentation (such as region growing, thresholding, region-based level sets), since such methods are sensitive to the spurious intensity variations and methods on inhomogeneity correction are required [23].

### Manual Breast Density Estimation

**Figure 4:**
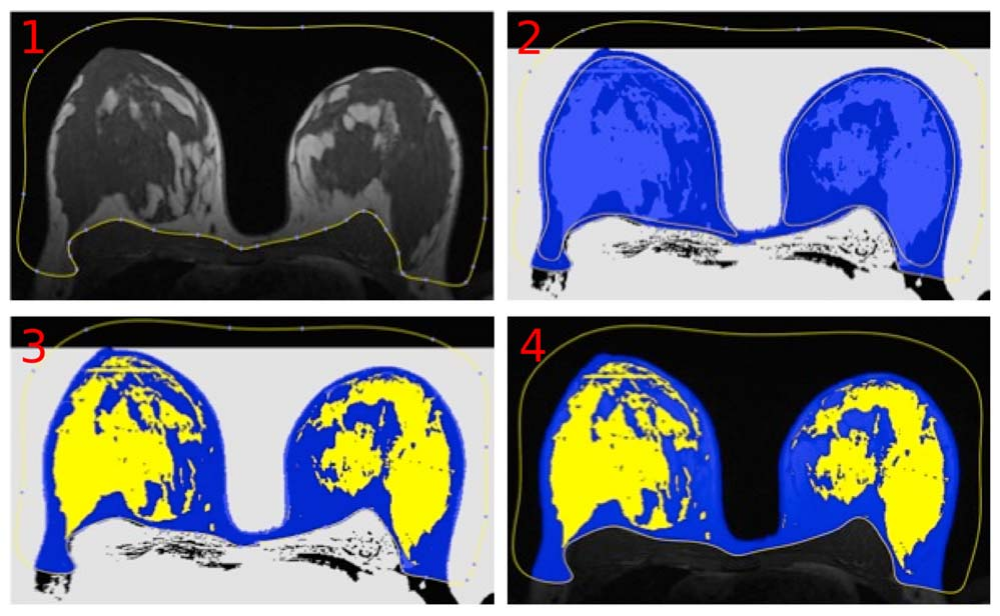
Steps of manual breast segmentation. In each slice, the user first marks the breast boundary and then detects the parenchymal regions.

Manual breast density measurements were performed in a free DICOM-Viewer (OsiriX version 3.8.1) by using a self designed plug-in. The manual measurements were carried out slice-by-slice on the unenhanced non-fat-suppressed 3D T1-weighted TWIST sequence. The steps of manual processing are shown in Figure 4. First, an experienced radiologist created a region of interest (ROI) including both breasts and separated the breast from the breast muscle and the background. The physiological landmark for the breast-body cut was the posterior boundary of the sternum. Second, all voxels within the ROI with a signal intensity (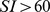) were collected in a brush-ROI automatically by the plug-in. Thereafter, both breasts were manually processed to include all fibroglandular tissue in each breast. Finally, black and white masks of the total breast as well as the parenchymal tissue were created, the volumes were calculated slice-by-slice and saved into a database.

Our experienced radiologists required about an hour to manually delineate the breast tissue in all slices of a single dataset. This procedure is rather straightforward for the user, since the breast, as well as the boundaries, are clearly visible. The segmentation of the fibroglandular tissue, which is a rather sparse structure without any distinguishable pattern, is a challenge even for an experienced reader. The manual delineation of the parenchyma in one dataset in some cases took dozens of working hours.

### Automated Breast Density Estimation

**Figure 5:**
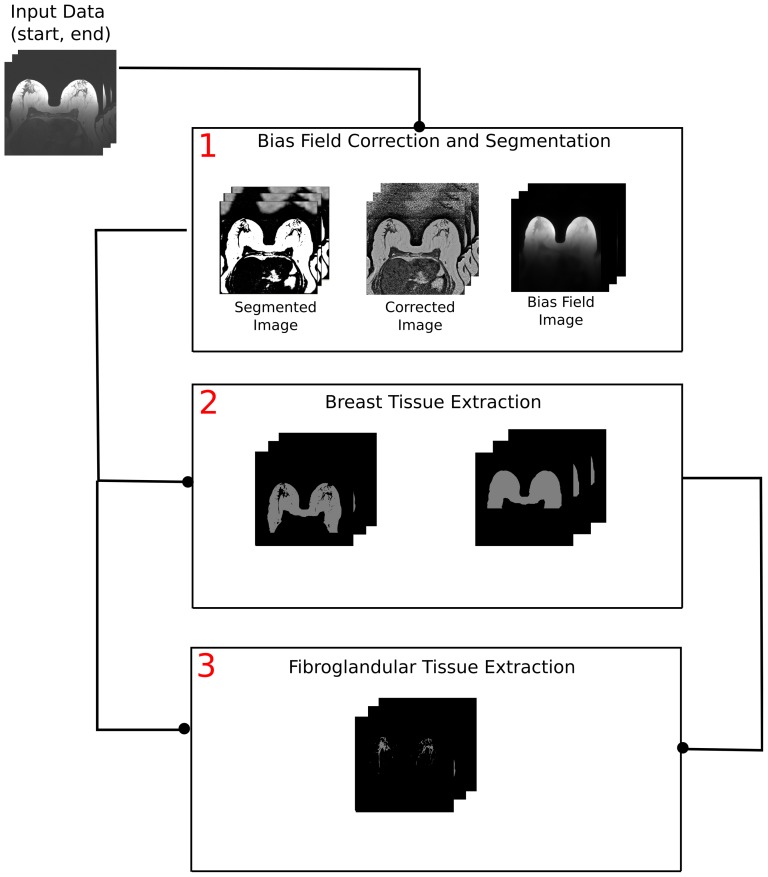
An overview of the automated breast density evaluation framework. The approach consists of three main steps: segmentation and bias field correction, breast tissue delineation, and fibroglandular tissue extraction. The results of the bias field correction (Step 1) are used both for breast tissue (Step 2) and parenchyma (Step 3) extraction. In Step 3, the results of Steps 1 and 2 are utilized for parenchyma extraction. The data flow is schematically explained by the lines, connecting the pipeline steps.

The automated processing is carried out on the unenhanced non-fat-suppressed 3D T1-weighted TWIST sequence. The framework for automated breast density evaluation consists of three main procedures. A general overview of the segmentation pipeline is shown in Figure 5. The user selects the start and end slices for the processing to avoid irrelevant computations as well as pre-selects the parameters for further processing steps. Usually, no intermediate parameter triggering is required.

First, the recently developed algorithm for simultaneous segmentation and bias field correction [19] is applied to the selected slices. In the algorithm, the classical image model is used: a 2D image 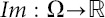 is defined on a continuous domain 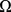, such that 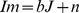, where 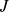 is the true image, 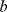 is the slowly varying intensity inhomogeneity component, and 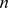 is the additive zero-mean Gaussian noise. Using this model and the assumptions about 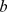, we formulated a clustering energy and converted it to the variational two-phase level set functional, which is then minimized with respect to its variables. The details of the procedure are given in [19]. The algorithm processes each 2D slice and composes the results back into a 3D volume. For each slice, three result images are produced: a breast and parenchyma segmented image, an inhomogeneities corrected image, and a bias field image.

Second, a boundary refinement procedure is executed. Here, a 3D breast connected component (as the biggest 3D connected component) is selected and extracted from the segmented image, obtained in the first step. Its intensity is set 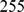. The background intensity equals to 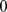. Thereafter, two characteristics of the breast component are computed:

- *the center of masses* of the breast component is denoted as the point with coordinates 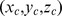;
- *the minimal axis-aligned bounding box* is defined by the upper left corner point with coordinates 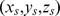 and the lower right corner point with coordinates 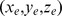.

The slices, which presumably have a correctly segmented breast-pectoral muscle boundary, are found. We look for slices, which do not differ significantly from their closest neighbours in 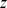 direction. Starting from slice 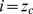, we select every consecutive triple of slices 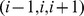 in both directions, namely for 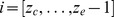 and 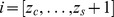. Let 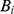, 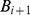, and 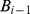 be the breast components in slices 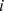, 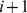, and 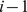, correspondingly. We compute and analyse the 2D connected components 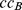 in two slices 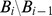 and 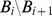, where the symbol 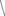 denotes set difference. The connected components 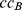 may have either positive, or negative intensities. If the maximal size of 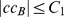, where 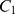 is a constant parameter and 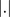 denotes the area, then the breast component in slice 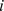 is similar to the breast components in slices 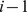 and 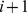. This slice is set as the reference 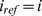. This procedure finishes, when at least one such slice is found. Often, the slice 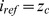 is selected as the reference one for both directions.

Thereafter, the boundary separation procedure starts. The procedure is described in pseudo-code:

**for** i: = 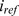 to 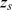
+1 **do**

begin

{

j  =  i-1;

evaluateComponents (i,j);

updateSlice (j);

}

end;

**for** i: = 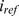 to 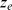-1 **do**

begin

{

j  =  i+1;

evaluateComponents (i,j);

updateSlice (j);

}

end;

The procedure 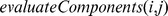 consists of the following steps:

- In slice 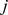 2D Distance Transformation [24] for the breast component is computed. The distance map is then inverted.
- In slices 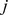 2D Watershed [24] on the inverted distance map is computed.
- Every connected component 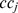, computed with the Watershed in slice 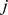, is overlaid with the breast connected component 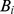 in slice 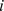, and the area of overlap is computed.
- Only components with significant overlap (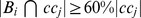) are kept. The rest is discarded.

In the procedure 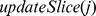, the intensities of the collected connected components and the boundaries between them are set to 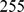, the rest is set to 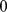.

**Figure 6:**
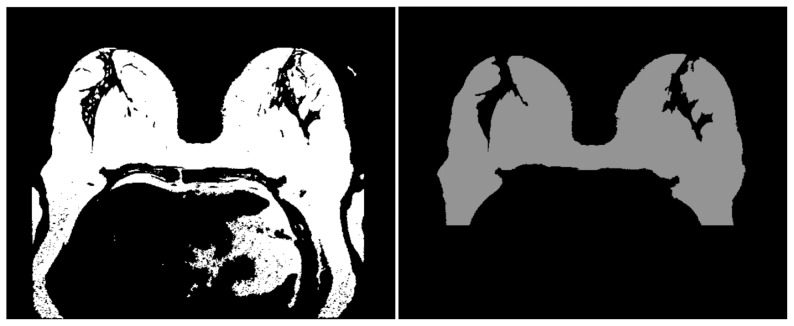
Intermediate steps of the postprocessing for breast tissue extraction. Left: a segmentation result with the breast tissue connected to the pectoral muscle. Right: the segmentation is corrected and the minor holes are closed as well. The lower part of the breast image is excluded from further postprocessing.

With this procedure, the boundary from the slice 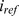 is propagated to the other slices, and the significant oversegmentations are excluded. Results of this procedure are shown in Figure 6. In the left column the breast tissue, which leaked into the pectoral muscle boundary, is presented. The correction result is depicted in the right column. Additionally, minor holes are closed with the Hole Filling filter [25], and the lower part of the breast image (the pixels that have y coordinates in 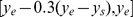), which contains irrelevant structures (such as lung parts), is automatically excluded.

Thereafter, the concavities in the 3D breast tissue are closed using morphological operations, namely, with the Rolling Ball filter [24]. Here, several filters with different radii are applied, dependent on the breast location. The top of the breasts, where the concavities are usually defined by the nipple and areola regions, the rolling ball filter with the kernel size 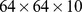 is applied. These locations are found as the breast tissue pixels with y coordinates in 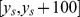.

Then, the breast regions that are adjacent the pectoral muscle boundary are detected. These are the regions that have the lowest background component in the neighbourhood 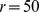, and the y coordinates of these pixels are close to 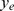. Here, the concavities are processed with the kernel size 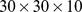.

The rest of the breast (untouched by other filters), which includes the breast sides and skin folds, is processed with the small kernel size 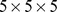.

**Figure 7:**
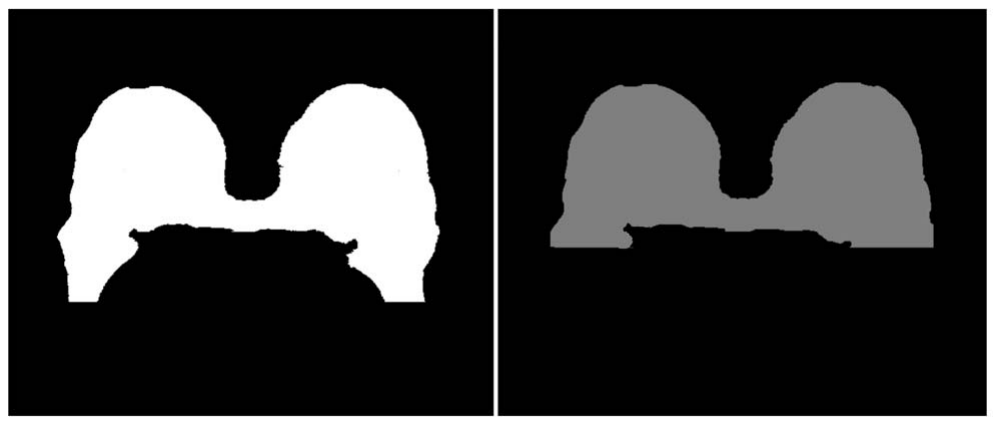
Final steps for breast tissue extraction. Left: the concavities of the fibroglandular tissue are closed with morphological operations. Right: the approximate location of the sternum bone is computed and the cut is done along its lower boundary.

The approximate position of the sternum is automatically computed. The sternum detection procedure is based on the general breast geometry. The lowest breast tissue point 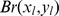, which is located close to the slice center, denotes the upper sternum boundary. The pixels with y coordinates that lie below 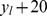, which indicates the posterior boundary of the sternum, are removed from the segmented image. The results are shown in Figure 7.

Third, the fibroglandular tissue regions are extracted from the segmented images, which were previously computed. The image of the extracted 3D breast, computed in the second processing step, is used as a mask. Let us denote 3D extracted breast image as 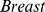. The segmented image is denoted as 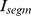. The mask operation 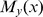 removes all regions in 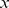, where 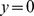. The fibroglandular tissue region 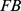 is defined as the difference between the masked segmented image and the extracted 3D breast, i. e. 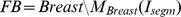.

Finally, the BV and PV values for the total dataset are computed from the black-and-white mask images as the number of non-zero pixels in the breast and fibroglandular tissue regions, correspondingly (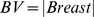 and 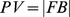).

### Statistical Analysis

Data are given as mean and standard deviation (SD). To compare the results of the automated algorithm with the manual results produced by the radiology experts the segmentation accuracy was measured by Dice's Similarity Coefficient (DSC) [26]. Additionally, we utilized delineation sensitivity and specificity metrics [27]. Let 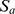 and 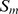 be two segmentation results, namely, the one provided by the automatic method and the one provided by the expert manual readings, correspondingly. The Dice coefficient is computed as follows: 
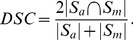
(1)


Udupa et al. [27] proposed to define the delineation sensitivity (or true positive volume fraction, which indicates the fraction of the total amount of tissue in the reference segmentation that was correctly identified by the automatic method) and delineation specificity (or true negative volume fraction, which describes the fraction of the total amount of tissue in the reference region 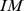 that is truly not in the object) as: 
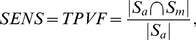
(2)

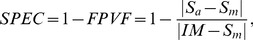
(3)where 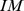 is the whole image and 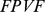 is the false positive volume fraction.

Bland-Altman plots [28] analysed the statistical agreement between the manual and automatic volume assessment methods. The plot displays the difference between the measures against their average, i. e. for each subject 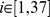 the point in the Bland-Altman plot is computed as 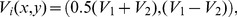
(4)where 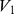 and 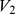 are two volume values (the automatic and manual results) for the subject 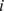. The plot allows one to investigate the existence of any systematic difference between the measurements and to identify possible outliers. The average difference (mean bias) is the estimated bias, which is computed as the value determined by one method minus the value determined by the other method, and the SD of the differences measures the random fluctuations around this mean.

In addition to Blan-Altman plots, we also built simple linear regression [29] plots for volume values.

## Results

The datasets were processed on an Intel(R) Core i7-950 @ 3.06 GHz computer with 6 GB DDR3 RAM. The first processing step was implemented on an NVIDIA parallel computing platform (CUDA) and the computations were run on NVIDIA Tesla C2070. The refinement procedure was implemented in C++ within MeVisLab platform [30].

**Figure 8:**
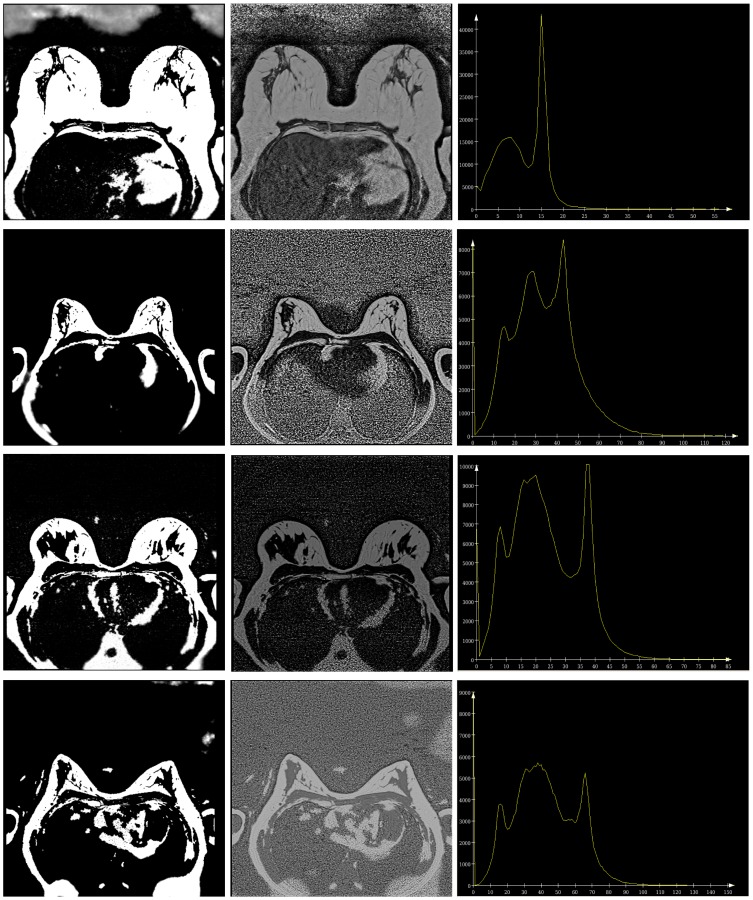
Results for slices, shown in **Figure 1**. Left column: segmented images; middle column: corrected images; right column: histograms of the corrected images. The breast tissue becomes homogeneous after the correction. The histograms show the clearly separated intensity classes.

Since our data exhibit significant intensity inhomogeneities, the efficient intensity inhomogeneity correction is the central step in the pipeline. In Figure 8, the results of the segmented breast and the intensity correction, as well as the histogram of the corrected images are shown. One can observe that after processing the entire breast looks homogeneous with no smooth intensity variations in different locations of the same tissue. The histograms of the bias field corrected images are multimodal, and the corrected images can be properly segmented into several classes even with basic image processing techniques, for example, thresholding.

The total processing time for one dataset was about one hour, including postprocessing and saving procedures. The parameter values for the first processing step were optimized in terms of convergence speed for each ACR group. In the steps 2 and 3, the parameters were preselected and fixed for all datasets without additional optimization. In general, the optimal parameter selection is a trial-and-error process, and it is expected that for other breast sequences some prior parameter adaptation will be required.

**Figure 9:**
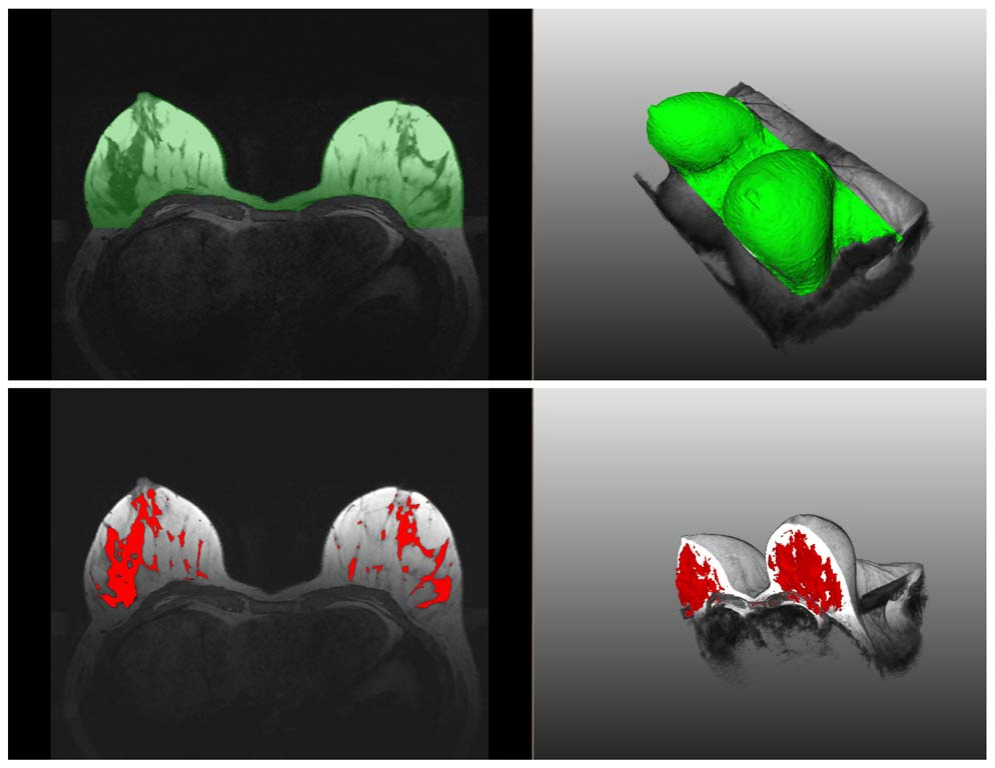
An example of breast tissue (upper row) and fibroglandular tissue (lower row) segmentation results. The results are overlaid in 2D and 3D with the original data.

**Figure 10:**
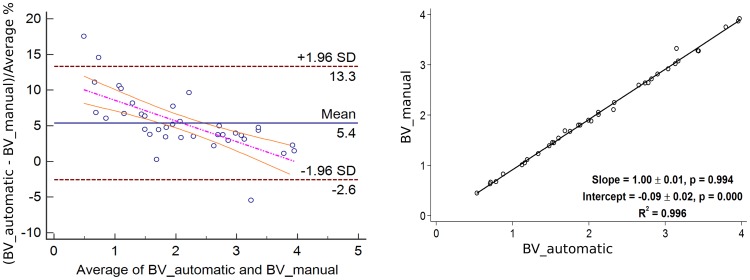
Bland-Altman with the regression line of differences on average (left) and linear regression (right) plots for the Breast Volume (BV) values of 37 datasets. The agreement is slightly higher for bigger breasts.

**Figure 11:**
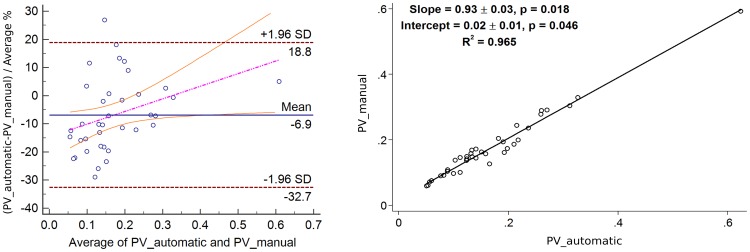
Bland-Altman with the regression line of differences on average (left) and linear regression (right) plots for the Parenchyma Volume (PV) values of 37 datasets. No exact influence of the breast density on the agreement results is observed.

In Figure 9, the segmentation results are shown in an overlaid 2D and 3D manner with the initial data. The result mean values of the Dice's Similarity Coefficients for BV and PV are given in Table 1 and Table 2, respectively (see also Table S1 and Table S2). The average DSC for BV of all 37 datasets equals 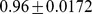. The average DSC for PV of all 37 datasets equals 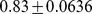. The Bland-Altman plots as well as the linear regression plots for BV and PV are presented in Figures 10 and 11. In Table 3, the values of mean bias obtained for each ACR group separately are presented. The mean bias for the breast volume computations is 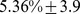. The mean bias for the parenchyma volume computations is 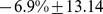. The difference of two methods regressed on the average of two methods is presented by the following regression equations: 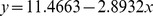 and 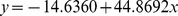 for BV and PV measurements, respectively. The information on regression coefficients is given in Table 4.

**Table 1:** Dice's Similarity Coefficients, Delineation Sensitivity, and Specificity for Breast Volumes (BV).

ACR	Mean 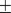 SD DSC	Mean 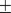 SD Sens	Mean 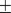 SD Spec
1	0.9715 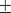 0.0026	0.985 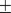 0.004	0.9921 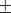 0.001
2	0.9637 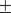 0.0154	0.977 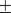 0.02	0.99436 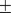 0.002
3	0.95367 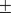 0.012	0.98 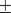 0.009	0.99 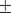 0.003
4	0.9469 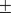 0.0244	0.987 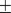 0.008	0.993 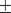 0.002
Total	0.96 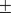 0.0172	0.9836 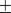 0.005	0.99 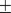 0.002

Breast Volume: Mean and standard deviation (SD) values of Dice's Similarity Coefficients (DSC), Sensitivity (Sens), and Specificity (Spec) for different ACR groups.

**Table 2:** Dice's Similarity Coefficients, Sensitivity, and Specificity for Parenchyma Volumes (PV).

ACR	Mean 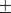 SD DSC	Mean 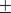 SD Sens	Mean 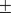 SD Spec
1	0.7637 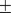 0.05	0.74 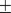 0.05	0.99 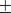 0.002
2	0.82613 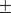 0.043	0.82 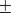 0.07	0.989 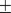 0.0003
3	0.8545 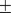 0.0549	0.82 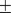 0.06	0.9927 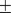 0.003
4	0.8798 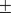 0.047	0.865 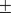 0.057	0.993 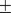 0.0044
Total	0.83 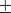 0.0636	0.81 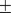 0.07	0.99 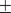 0.04

Parenchyma Volume: Mean and standard deviation (SD) values of Dice's Similarity Coefficients (DSC), Sensitivity (Sens), and Specificity (Spec) for different ACR groups.

**Table 3:** Breast and Parenchyma Mean Bias 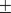 SD.

ACR	BV: Mean Bias ( 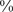 ) 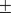 SD	PV: Mean Bias ( 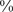 ) 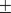 SD
1	3.33 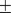 0.75	−8.1 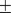 17.5
2	2.89 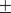 4.28	−1.929 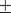 14.46
3	6.81 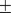 1.89	−10.39 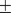 10.86
4	8.79 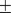 4.34	−7.5 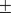 7.8
Mean	5.36 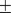 3.9	−6.9 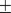 13.14

BV and PV Mean Bias (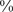) 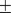 SD for different ACR groups.

**Table 4:** Coefficients of the regression lines of the differences.

Measurements	Coefficient	Value	P	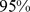 CI
BV	Intercept	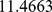	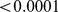	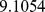 to 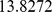
BV	Slope	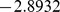	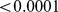	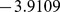 to 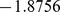
PV	Intercept	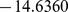	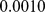	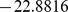 to 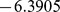
PV	Slope	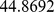	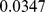	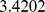 to 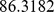

Coefficients of the regression lines of the difference between the two methods on the average of the two methods for BV and PV.

## Discussion

It can be observed in Tables 1 and 3, and Figure 10, the agreement between the automatic and manual breast segmentation methods is slightly better for bigger breasts. For example, in ACR 1 group, which usually consists of rather big breast with high percentage of fat, the mean Dice's similarity coefficient is 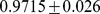, whereas in ACR 4 group, which consists of smaller dense breasts, the mean Dice's coefficient is 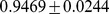. The Bland-Altman plot in Figure 10 also supports these observations. To evaluate this relationship formally, the difference between the volumes was regressed on the average of the volumes and the more accurate (regression-based) limits were calculated, as recommended by Bland and Altman [31]. The linear regression equation for the difference between the volumes on the averaged volumes is 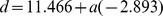, where 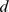 denotes the differences and 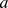 is the averages. The relation is statistically highly significant with 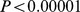. The regression of the absolute residual values on the averages gives the following equation: 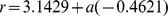. Finally, the formula for the more accurate 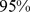 limits of agreement is: 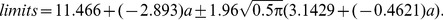
(5)


The agreement for the parenchyma detection (cf. Table 2 and Figure 11) has the opposite trend, i.e. the agreement rates that are achieved for ACR 1 group (the average DSC is 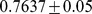) are lower than the rates, achieved for ACR 4 group (the average DSC is 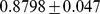), which is explained by the amount of parenchyma in the breast tissue, especially in the breasts with high percentage of fat. The obtained Dice's coefficients' behaviour is intuitively well explained by the definition of this metric itself. Such a trend is defined by the portion of coinciding voxels, where the manual and automatic breast (or parenchyma) masks are overlaid: the bigger these portions, the higher the coefficient values are. Therefore, the Dice's coefficients for BV are slightly decreasing and the Dice's coefficients for PV are increasing from ACR 1 to ACR 4. The Bland-Altman plots, especially for PV, constructed for each ACR group show no exact influence of the breast density on the results (cf. Table 3), i. e., no significant trend in mean biases can be observed. The linear regression equation for the difference between the volumes on the averaged volumes is 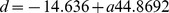 with 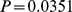, so no regression-based limits of agreement will be computed.

The sensitivity coefficients given in Tables 1 and 2, as expected, are rather close to the Dice's coefficients and have a similar trend, which is clearly observable for PV values. The specificity values are close to 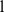, which shows that the automatic method does not produce a high amount of false positives within the image domain. However, the delineation specificity does not seem to be a reliable measure, since it will be always close to 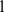, if the reference region, i. e. 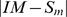, is significantly larger than any possible oversegmentation produced by 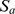.

Compared to previous works, the DSC values obtained by the proposed automated framework for the breast and parenchyma volumes (average BV: 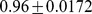; PV: 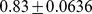) are higher than the ones in other works. For the breast tissue: Gallego-Ortiz et al. [17] and Gubern-Merida et al. [16] reported an average DSC value of 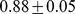 and 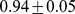, respectively. For breast parenchyma: Gubern-Merida et at. [16] and Wu et al. [18] reported an average DSC value of 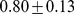, and 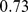, respectively. However, it should be mentioned that these comparisons are not fully adequate, since they were evaluated on different datasets. The way, how the data for the test sets were selected, as well as different vendors and sequence types, may have strong influences on results.

**Figure 12:**
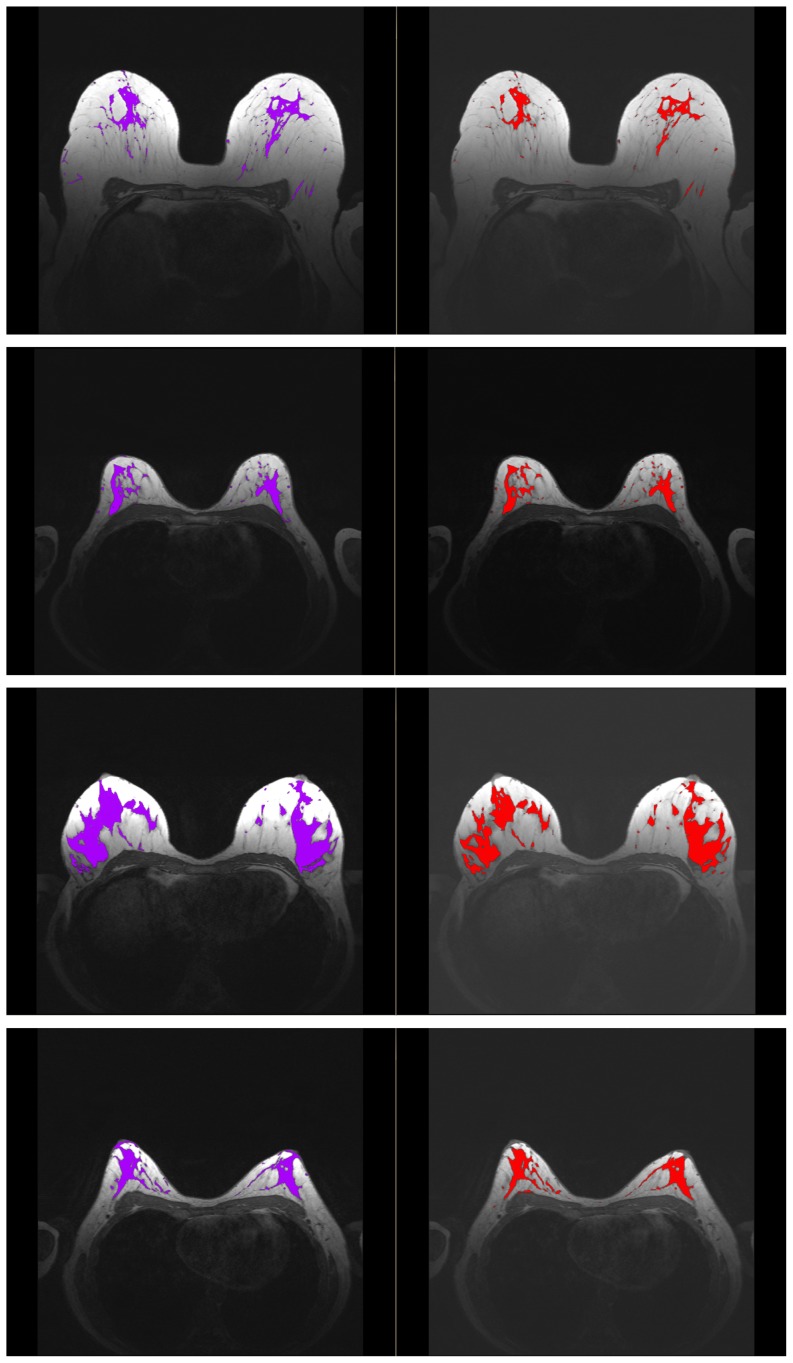
Manual (purple) and automatic (red) parenchyma segmentation results for four datasets. Whereas the results are very similar, one can observe that the user selects usually more than the algorithm.

The comparison of the automatic and manual segmentation results shows strong agreement for BV and sufficient agreement PV. In Figure 12, several results for the manual and automatic parenchyma segmentation are shown next to each other. Whereas the segmentation results look similar, one can observe that the user usually delineates more parenchymal tissue than the automated procedure. We plan to investigate this issue in more detail in future work as well as conduct an inter- and intra-observer variability study on parenchyma segmentation.

## Conclusions

Our study showed that the automatic results strongly agree with the manual segmentation and volumetry. Therefore, the proposed framework for automated breast density evaluation has high potential to be applied in the clinical and epidemiological routine to process numerous participant data.

## Supporting Information

Table S1 **Breast Volume (BV) Values, Dice's Coefficients, Sensitivity and Specificity Values for 37 Datasets.** Volume values produced by the user (Manual) and the automatic algorithm (Auto) are given in liters (L) and voxels (Vx).
(PDF)

Table S2 **Parenchyma Volume (PV) Values, Dice's Coefficients, Sensitivity and Specificity Values for 37 Datasets.** Volume values produced by the user (Manual) and the automatic algorithm (Auto) are given in liters (L) and voxels (Vx).
(PDF)

## References

[1] McCormack VA, dos Santos Silva I. Breast density and parenchymal patterns as markers of breast cancer risk: a meta-analysis. Cancer Epidemiology Biomarkers & Prevention. 2006;15:1159–1169. 10.1158/1055-9965.EPI-06-0034 16775176

[2] Byrne C, Schairer C, Wolfe J, Parekh N, Salane M, et al Mammographic features and breast cancer risk: effects with time, age, and menopause status. Journal of the National Cancer Institute. 1995;87:1622–1629. 7563205 10.1093/jnci/87.21.1622

[3] Boyd NF, Guo H, Martin LJ, Sun L, Stone J, et al Mammographic density and the risk and detection of breast cancer. New England Journal of Medicine. 2007;356:227–236. 17229950 10.1056/NEJMoa062790

[4] Kato I, Beinart C, Bleich A, Su S, Kim M, et al A nested case-control study of mammographic patterns, breast volume, and breast cancer (new york city, ny, united states). Cancer Causes & Control. 1995;6:431–438. 8547541 10.1007/BF00052183

[5] Saftlas AF, Hoover RN, Brinton LA, Szklo M, Olson DR, et al Mammographic densities and risk of breast cancer. Cancer. 1991;67:2833–2838. 2025849 10.1002/1097-0142(19910601)67:11<2833::aid-cncr2820671121>3.0.co;2-u

[6] Torres-Mejía G, De Stavola B, Allen DS, Pérez-Gavilán JJ, Ferreira JM, et al Mammographic features and subsequent risk of breast cancer: a comparison of qualitative and quantitative evaluations in the guernsey prospective studies. Cancer Epidemiology Biomarkers & Prevention. 2005;14:1052–1059. 10.1158/1055-9965.EPI-04-0717 15894652

[7] Van Gils C, Hendriks J, Otten J, Holland R, Verbeek A. Parity and mammographic breast density in relation to breast cancer risk: indication of interaction. European Journal of Cancer Prevention. 2000;9:105–112. 10830577 10.1097/00008469-200004000-00006

[8] Thomas DB, Carter RA, Bush WH, Ray RM, Stanford JL, et al Risk of subsequent breast cancer in relation to characteristics of screening mammograms from women less than 50 years of age. Cancer Epidemiology Biomarkers & Prevention. 2002;11:565–571. 12050098

[9] Maskarinec G, Pagano I, Lurie G, Kolonel LN. A longitudinal investigation of mammographic density: the multiethnic cohort. Cancer Epidemiology Biomarkers & Prevention. 2006;15:732–739. 10.1158/1055-9965.EPI-05-0798 16614116

[10] Boyd N, Byng J, Jong R, Fishell E, Little L, et al Quantitative classification of mammographic densities and breast cancer risk: results from the canadian national breast screening study. Journal of the National Cancer Institute. 1995;87:670–675. 7752271 10.1093/jnci/87.9.670

[11] Balleyguier C, Ayadi S, Van Nguyen K, Vanel D, Dromain C, et al BIRADS classification in mammography. European journal of radiology. 2007;61:192–194. 17164080 10.1016/j.ejrad.2006.08.033

[12] Klifa C, Carballido-Gamio J, Wilmes L, Laprie A, Lobo C, et al. Quantification of breast tissue index from mr data using fuzzy clustering. In: Engineering in Medicine and Biology Society, 2004. IEMBS′04. 26th Annual International Conference of the IEEE. IEEE. 2004; volume 1, pp.1667–1670. 10.1109/IEMBS.2004.1403503 17272023

[13] Lin M, Chan S, Chen JH, Chang D, Nie K, et al A new bias field correction method combining n3 and fcm for improved segmentation of breast density on mria). Medical physics. 2011;38:5–14. 21361169 10.1118/1.3519869 PMC3017578

[14] Nie K, Chen JH, Chan S, Chau MKI, Hon JY, et al Development of a quantitative method for analysis of breast density based on three-dimensional breast mri. Medical physics. 2008;35:5253–5262. 19175084 10.1118/1.3002306 PMC2673600

[15] Wang L, Platel B, Ivanovskaya T, Harz M, Hahn HK. Fully automatic breast segmentation in 3d breast mri. In: Biomedical Imaging (ISBI), 2012 9th IEEE International Symposium on. IEEE. 2012; pp.1024–1027.

[16] Gubern-Mérida A, Kallenberg M, Mann RM, Martı R, Karrsemeijer N. Breast segmentation and density estimation in breast mri: A fully automatic framework. IEEE J of Biomedical and Health Informatics. 2014. 10.1109/JBHI.2014.2311163 25561456

[17] Ortiz CG, Martel A. Automatic atlas-based segmentation of the breast in mri for 3d breast volume computation. Medical physics. 2012;39:5835–5848. 23039622 10.1118/1.4748504

[18] Wu S, Weinstein SP, Conant EF, Kontos D. Automated fibroglandular tissue segmentation and volumetric density estimation in breast mri using an atlas-aided fuzzy c-means method. Medical physics. 2013;40:122302. 24320533 10.1118/1.4829496 PMC3852242

[19] Ivanovska T, Wang L, Laqua R, Hegenscheid K, Volzke H, et al. A fast global variational bias field correction method for mr images. In: Image and Signal Processing and Analysis (ISPA), 2013 8th International Symposium on. IEEE. 2013; pp.667–671.

[20] Völzke H, Alte D, Schmidt CO, Radke D, Lorbeer R, et al. Cohort profile: the study of health in pomerania. International journal of epidemiology: 2010;dyp394. 10.1093/ije/dyp394 20167617

[21] Hegenscheid K, Kühn J, Völzke H, Biffar R, Hosten N, et al. Whole-body magnetic resonance imaging of healthy volunteers: pilot study results from the population-based ship study. In: RöFo-Fortschritte auf dem Gebiet der Röntgenstrahlen und der bildgebenden Verfahren. © Georg Thieme Verlag KG Stuttgart· New York. 2009; volume 181, pp.748–759. 10.1055/s-0028-1109510 19598074

[22] Kuhl CK, Kooijman H, Gieseke J, Schild HH. Effect of b1 inhomogeneity on breast mr imaging at 3.0 t. Radiology. 2007;244:929–930. 17709843 10.1148/radiol.2443070266

[23] Vovk U, Pernus F, Likar B. A review of methods for correction of intensity inhomogeneity in mri. Medical Imaging, IEEE Transactions on. 2007;26:405–421. 10.1109/TMI.2006.891486 17354645

[24] Soille P. Morphological image analysis: principles and applications. Springer-Verlag New York, Inc. 2003.

[25] Gonzales RC, Woods RE. Digital image processing, 2-nd edition. 2002.

[26] Dice LR. Measures of the amount of ecologic association between species. Ecology. 1945;26:297–302.

[27] Udupa JK, Leblanc VR, Zhuge Y, Imielinska C, Schmidt H, et al A framework for evaluating image segmentation algorithms. Computerized Medical Imaging and Graphics. 2006;30:75–87. 16584976 10.1016/j.compmedimag.2005.12.001

[28] Bland JM, Altman DG. Statistical methods for assessing agreement between two methods of clinical measurement. The lancet. 1986;327:307–310. 2868172

[29] Montgomery DC, Peck EA, Vining GG. Introduction to linear regression analysis, volume 821. John Wiley & Sons. 2012.

[30] Ritter F, Boskamp T, Homeyer A, Laue H, Schwier M, et al Medical image analysis. Pulse, IEEE. 2011;2:60–70. 10.1109/MPUL.2011.942929 22147070

[31] Bland JM, Altman DG. Measuring agreement in method comparison studies. Statistical methods in medical research. 1999;8:135–160. 10501650 10.1177/096228029900800204

