# Supplementary material for: A Level Set Based Framework for Quantitative Evaluation of Breast Tissue Density from MRI Data
Source: PLoS One. 2014 Nov 25;9(11):e112709. doi: 10.1371/journal.pone.0112709 (PMC4244105; doi:10.1371/journal.pone.0112709)
Supplement: Table S1 — Breast Volume (BV) Values, Dice's Coefficients, Sensitivity and Specificity Values for 37 Datasets. Volume values produced by the user (Manual) and the automatic algorithm (Auto) are given in liters (L) and voxels (Vx). (PDF) [file pone.0112709.s001.pdf]

Table **S1**: Breast Volume (BV) Values, DICE Coefficients, Sensitivity and Specificity Values for 37 Datasets.

ACR	DICE	Sensitivity	Specificity	BV Auto (L)	BV Manual (L)	BV Auto (Vx)	BV Manual (Vx)
1	0.9714	0.99	0.99	3.03836	2.92033	4593358	4414930
1	0.97	0.98	0.993	2.73837	2.63748	4139839	3987315
1	0.9711	0.98	0.9932	2.82172	2.71754	4265843	4108356
1	0.9691	0.9844	0.9924	3.17231	3.07488	4795867	4648566
1	0.9721	0.9866	0.9937	2.89959	2.81567	4383568	4256700
1	0.9711	0.9928	0.99	3.42862	3.28158	5183342	4961065
1	0.9703	0.988	0.99	3.131	3.01861	4733409	4563501
1	0.97796	0.98875	0.9955	2.652	2.5947	4009281	3922643
1	0.9705	0.9817	0.99125	3.9586	3.86876	5984563	5848746
2	0.9677	0.9913	0.9949	1.88665	1.79873	2852207	2719307
2	0.9394	0.9947	0.996	0.705045	0.630705	1065879	953493
2	0.96771	0.969	0.9972	1.688	1.68292	2552041	2544221
2	0.97457	0.9911	0.9956	2.12202	2.05231	3208039	3102668
2	0.97623	0.9938	0.9952	2.33139	2.24992	3524566	3401410
2	20.95887	0.98	0.9947	1.75293	1.67652	2650058	2534543
2	0.982433	0.98976	0.9946	3.97511	3.91665	6009525	5921153
2	0.97728	0.9943	0.9964	1.8618	1.79909	2814649	2719839
2	0.95276	0.9581	0.989	3.79382	3.75147	5735450	5671440
2	0.94	0.91	0.99	3.14845	3.32466	4759785	5026193
3	0.95	0.9839	0.9943	1.4811	1.38604	2239106	2095402
3	0.965	0.99	0.994	1.99603	1.89472	3017573	2864413
3	0.96748	0.98	0.996	1.52197	1.45497	2300892	2199610
3	0.963454	0.995	0.995	1.54088	1.44582	2329488	2185783
3	0.9334	0.9668	0.99	0.71128	0.664	1075309	1003491
3	0.94636	0.994	0.9887	2.32338	2.10967	3512465	3189383
3	0.9431	0.9766	0.99	2.0305	1.87862	3069679	2840075
3	0.95838	0.9886	0.9971	0.877	0.825312	1326461	1247699
3	0.9439	0.9968	0.99	1.12277	1.00958	1697396	1526279
3	0.965537	0.989	0.989	3.43654	3.27639	5195317	4953207
4	0.93	0.9788	0.9936	1.1585	1.04554	1751403	1580631
4	0.9	0.9788	0.99	0.533621	0.4474	806721	676375
4	0.9564	0.985	0.9957	1.33776	1.23249	2022412	1863270
4	0.963	0.9804	0.9948	2.12722	2.01113	3215900	3040391
4	0.9623	0.9908	0.9933	1.60144	1.54139	2421042	2330253
4	0.9715	0.996	0.992	2.77817	2.64294	4200005	3995566
4	0.9303	0.9978	0.9949	0.782	0.675684	1182252	1021492
4	0.9619	0.995	0.996	1.19106	1.11353	1800625	1683422
