# Supplementary material for: A Level Set Based Framework for Quantitative Evaluation of Breast Tissue Density from MRI Data
Source: PLoS One. 2014 Nov 25;9(11):e112709. doi: 10.1371/journal.pone.0112709 (PMC4244105; doi:10.1371/journal.pone.0112709)
Supplement: Table S2 — Parenchyma Volume (PV) Values, Dice's Coefficients, Sensitivity and Specificity Values for 37 Datasets. Volume values produced by the user (Manual) and the automatic algorithm (Auto) are given in liters (L) and voxels (Vx). (PDF) [file pone.0112709.s002.pdf]

Table **S2**: Parenchyma Volume (PV) Values, DICE Coefficients, Sensitivity and Specificity Values for 37 Datasets.

ACR	DICE	Sensitivity	Specificity	PV Auto (L)	PV Manual (L)	PV Auto (Vx)	PV Manual (Vx)
1	0.8075	0.74	0.99	0.10	0.14	155928	208291
1	0.77	0.73	0.99	0.08921	0.108815	134867	164506
1	0.73	0.84	0.99	0.165841	0.126567	250717	191342
1	0.754	0.7	0.99	0.076892	0.090123	116245	136247
1	0.684	0.65	0.99	0.053374	0.060495	80691	91457
1	0.81	0.75	0.99	0.140991	0.171569	213149	259376
1	0.79	0.77	0.99	0.140931	0.143835	213058	217448
1	0.8305	0.784	0.99	0.12416	0.141587	187704	214050
1	0.701	0.736	0.99	0.112409	0.100071	169939	151287
2	0.89	0.862	0.99	0.151374	0.162598	228845	245814
2	0.797	0.73	0.98	0.05103	0.059081	77147	89318
2	0.8425	0.89	0.98	0.198101	0.173339	299497	262052
2	0.8718	0.872	0.997	0.236933	0.235699	358193	356328
2	0.84	0.77	0.99	0.131694	0.158148	199094	239087
2	0.77	0.69	0.99	0.060042	0.074933	90771	113284
2	0.8	0.84	0.996	0.218066	0.199381	329669	301422
2	0.87	0.829	0.99	0.123936	0.1372	187365	207417
2	0.77	0.83	0.99	0.210567	0.186423	318332	281832
2	0.81	0.91	0.990	0.192847	0.160918	291544	243275
3	0.9	0.867	0.99	0.181921	0.203889	275026	308238
3	0.823	0.79	0.99	0.133425	0.168792	201710	255178
3	0.8596	0.81	0.99	0.124111	0.148652	187630	224731
3	0.898	0.8646	0.998	0.21562	0.24351	325972	368136
3	0.82	0.76	0.998	0.10007	0.096698	151284	146188
3	0.89	0.85	0.99	0.259655	0.27825	392543	420656
3	0.724	0.73	0.99	0.112279	0.145761	169742	220360
3	0.9	0.885	0.998	0.158559	0.15743	239708	238001
3	0.85	0.77	0.99	0.089088	0.103886	134683	157054
3	0.88	0.894	0.993	0.623841	0.59302	943115	896522
4	0.92	0.89	0.99	0.190652	0.19368	288226	292803
4	0.79	0.82	0.99	0.082406	0.091186	124581	137855
4	0.906	0.924	0.997	0.32666	0.328832	493850	497125
4	0.91	0.89	0.998	0.2707	0.290958	409241	439865
4	0.92	0.944	0.996	0.312128	0.303971	471871	459541
4	0.869	0.827	0.998	0.2607	0.289561	394123	437755
4	0.83	0.773	0.99	0.056643	0.070907	85632	107197
4	0.893	0.849	0.99	0.133848	0.148482	202350	224474
